# DRESS syndrome due to iodinated contrast media. A case report

**DOI:** 10.1186/s13223-023-00774-7

**Published:** 2023-02-27

**Authors:** G. Zambrano Ibarra, B. Noguerado Mellado, P. Tornero Molina, C. Cuevas Bravo, P. Rojas Pérez-Esquerra

**Affiliations:** Allergy Department, Hospital General Universitario Gregorio Marañón, Calle Dr. Esquerdo 46, 28007 Madrid, Spain

**Keywords:** Non-inmediate hypersensitivity reactions, Drug reaction with eosinophilia and systemic symptoms, Iodinated contrast media, Intradermal tests

## Abstract

**Background:**

The most frequent non-immediate reactions described with iodinated contrast media (ICM) are mild to moderate, however, some cases of patients with severe non-immediate reactions, such as drug eruption with eosinophilia and systemic symptoms (DRESS) have been described.

**Case presentation:**

An 84-year-old patient developed DRESS syndrome after administration of ICM ioversol. The patient fullfilled the RegiSCAR diagnostic criteria for DRESS (definite score = 6). He underwent intradermal skin testing (IDT) with the widest panel of ICM available at our center. IDT was positive with ioversol and iomeprol. A punch biopsy was performed on the positive IDT with the culprit drug (ioversol) and histopathology was compatible with a T-cell mediated mechanism.

**Conclusion:**

In this case, the IDT-positive biopsy was consistent with DRESS syndrome caused by T-lymphocyte activation, supporting the clinical diagnosis.

## Background

Drug reaction with eosinophilia and systemic symptoms (DRESS), is potentially life-threatening severe cutaneous adverse drug reactions (SCARs). DRESS syndrome is characterized by generalized skin rash, fever, lymphadenopathy with at least one visceral involvement associated with hematological abnormalities mainly eosinophilia and lymphocytosis. Diagnostic criteria have been established by the European Registry of Serious Cutaneous Adverse Reactions (RegiSCAR) group [1].

The pathogenesis of DRESS syndrome has not been fully elucidated, although it has been proposed to be the result of drug exposure through a T-cell-mediated mechanism, genetic predisposition, and viral reactivation [1].

The most described non-inmediate hypersensitivity reactions (NIHR) to iodinated contrast media (ICM) are maculopapular exanthems and delayed urticaria of unclear mechanism. NIHR are reported to occur in 0.5–3% of patients receiving ICM, and may include severe life-threatening reactions, even though higher frequencies for NIHR have been reported [2, 3]. DRESS syndrome is not frequently caused by ICM, although some cases have been published, mainly in case reports [4–7], and recently a case series [8]. Skin testing is a useful tool for the diagnosis of hypersensitivity to ICM, although the predictive values in SCAR including DRESS are not known [9].

We report the case of a patient with DRESS syndrome after receiving an ICM, with sensitization demonstrated by skin testing and by punch biopsy on the positive skin test with the culprit ICM ioversol, confirming the mechanism involved in the reaction.

## Case presentation

An 84-year-old man with a personal history of prostate cancer with no other underlying medical conditions, underwent a CT scan with ICM ioversol in January 2021. No other suspected drugs were involved. He had no history of drug allergy and previously tolerated ICM.

Seven days later, he developed fever (temperature 38.7 °C) and an itchy erythematous, blanching morbilliform exanthema on the trunk that spread to the abdomen and upper limbs. Forty-eight hours later he went to the Emergency Department for persistent fever and skin worsening with facial edema and generalized maculopapular rash with intense erythematous-violaceous lesions on the face, trunk, back, chest and abdomen covering more than 60% of his body surface, without mucosal involvement or lymphadenopathy.

Blood test revealed an elevated cardiac enzyme level (troponin 41 ng/l), mild acute kidney injury (creatinine 1.29 mg/dl) and abnormal liver profile (GPT 108 U/L) that he did not have previously. He developed peripheral eosinophilia on the 4th day of admission, with peak eosinophil count of 2000 eos/uL.

Blood cultures were negative, and the C-reactive protein and antinuclear antibodies levels were within normal limits. Serologies were negative for HIV, HBV, HCV, EBV, CMV, syphilis and parvovirus.

The patient was admitted to the hospital and, after treatment with intravenous methylprednisolone, he had significant clinical and biochemical improvement.

Fever and rash were completely resolved whitin 15 days. On discharge, he had normalization of creatinine level (0.89 mg/dl), liver function (GPT 60 U/L), cardiac enzyme level (troponin 5.1 ng/l) and eosinophilia count (300 eos /uL).

On discharge, 5 more days of systemic corticosteroids were prescribed to complete the tapering.

Our patient’s clinical manifestations included an extensive dermatosis, fever, elevated cardiac enzyme levels, eosinophilia, acute renal and liver injury, without lymphadenopathy or mucosal involvement. Application of the RegiSCAR DRESS criteria in this case yielded a score of 7 (definitive) (skin involvement (≥ 50%) + 2, organ involvement + 2, eosinophilia: 2000 eos/uL. + 2, evaluation of other potential causes + 1) [1].

**Fig. 1:**
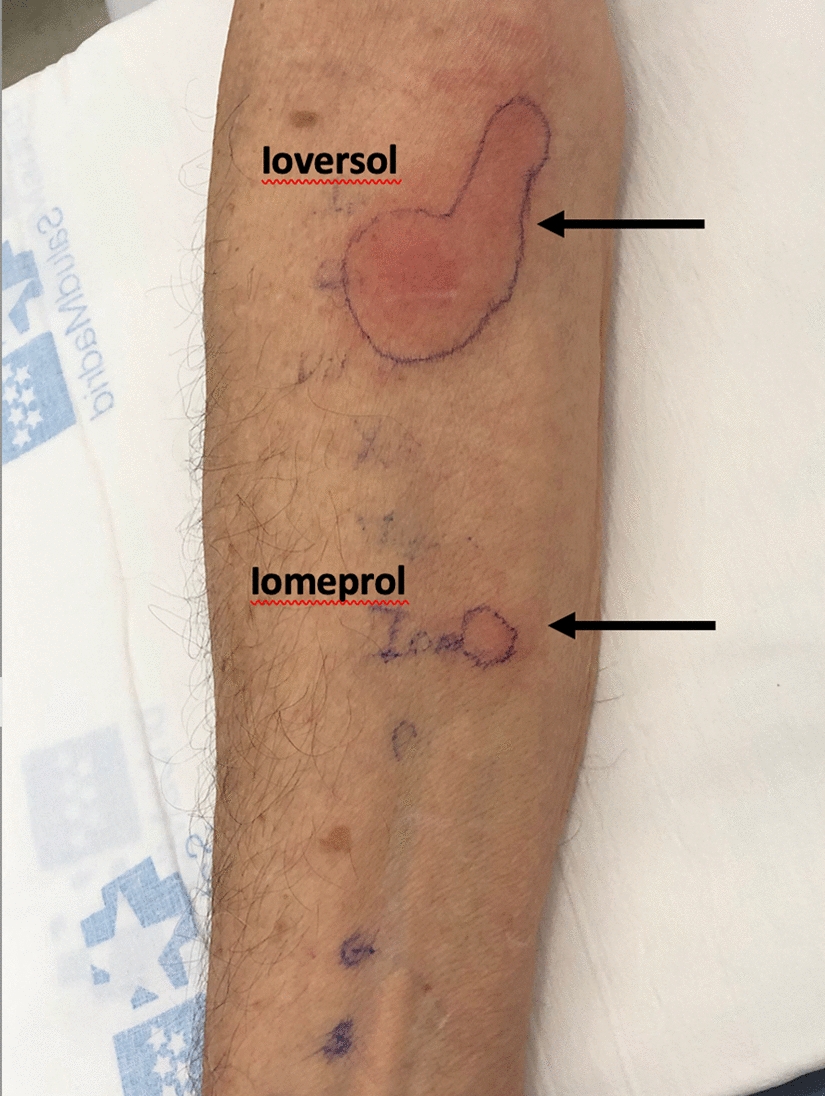
Intradermal test with contrast media. Image shows significant positivity to the culprit drug (ioversol) with cross-reactivity with iomeprol

Intradermal testing (IDT) with ioversol, and other ICM (diluted 1/10): iopamidol, iopramide, iobitridol, iodixanol, iomeprol were performed for etiologic diagnosis and possible cross-reactivity between ICM. Ioversol and iomeprol were positive at 24 h in late readings, and negative to the remaining ICM tested (Fig. 1).

**Fig. 2:**
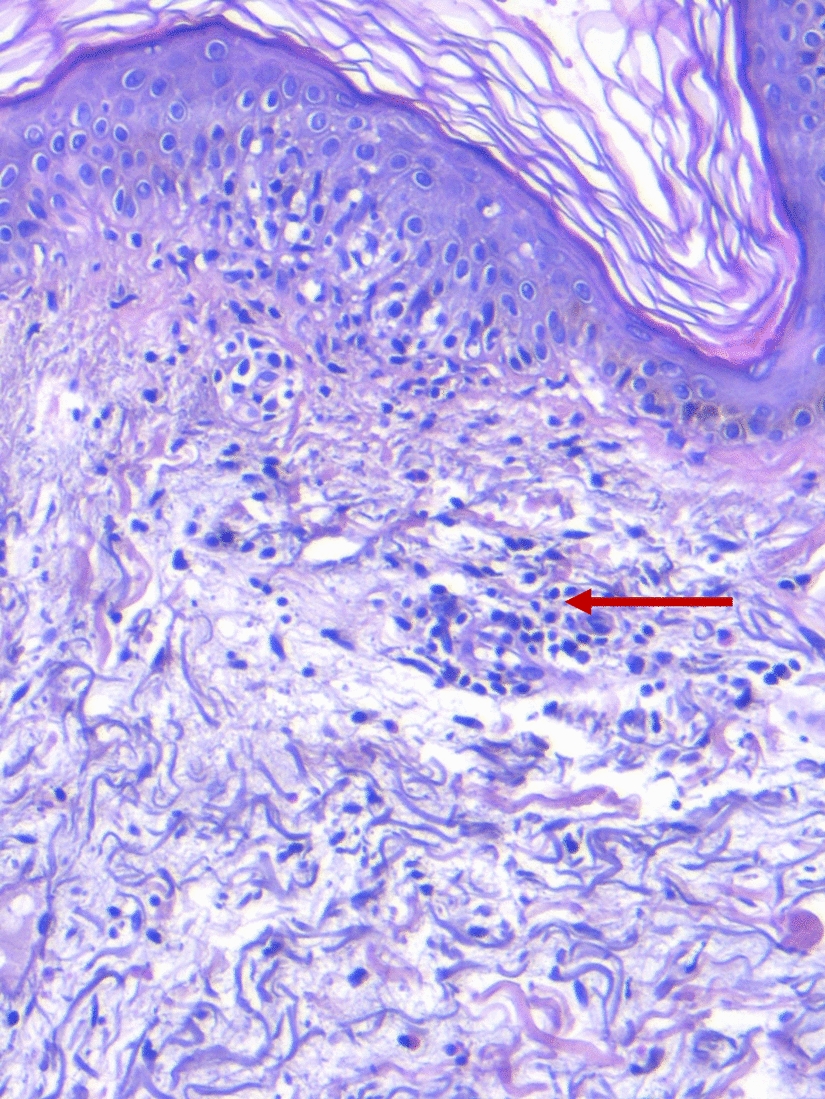
Histology of a skin biopsy performed on the positive intradermal test with the culprit drug (ioversol)

A punch biopsy was performed on the ioversol-positive IDT (Fig. 2) with dermatopathologic features suggestive of a drug reaction. Histology of the skin biopsy revealed a skin layer lined by epidermis of usual thickness and an orthokeratotic chorneal layer, without apoptotic keratinocytes or lichenoid inflammatory infiltrates. The underlying dermis showed a moderate superficial perivascular inflammatory infiltrate with lymphocytes and eosinophils, and epidermal involvement without microabscesses, micropustules or vesicles.

## Discussion

NIHR to ICM described are mainly mild to moderate. More severe skin reactions, including DRESS syndrome, have also been described, although less frequently. There are a few case reports in the literature of patients with DRESS syndrome to ICM, including iobiditrol [4], ioxaglate [4], ioxithalamate [5], ioversol [6], iohexol [4, 10] and iopramide [4]. Recently a French series reported 13 patients with DRESS syndrome to ICM: iohexol, ioversol, iobiditrol and iomeprol, showing cross-reactivity between ICM in approximately 77% of cases [8]. In NIHR, cross-reactions could be related to the presence of the carbamoyl side chain in some ICM [9].

There are published case series of patients with NIHR, evaluating the cross-reactivity of ICM based on skin testing [7], and the negative predictive value of skin testing for ICM [11], which included few patients with DRESS syndrome: 2 and 7 respectively. There is also a study that assess the delay of DRESS occurrence and culprit drugs, which included 5 patients with DRESS syndrome to ICM [12].

IDT with late reading and/or patch tests are useful for etiologic diagnostic confirmation and study of cross reactivity before administration of a new ICM. Some studies report variable negative predictive values for skin testing in NIHR to ICM; but the predictive values in DRESS are not well known [12].

We present a case of DRESS syndrome to ioversol with cross reactivity to iomeprol demonstrated by positive IDT. In this case, due to the severity of the reaction and comorbidities, we decided to avoid all ICM in the future.

A skin biopsy was not performed during the acute DRESS reaction, but due to a positive IDT with the suspected drug, it was decided to perform a skin biopsy on this positive IDT (ioversol) to demonstrate the presence of lymphocytes, which support the mechanism and diagnostic suspicion of DRESS syndrome.

In our case, the biopsy of the positive IDT with the culprit drug is confirmatory of the underlying immunological mechanism of DRESS syndrome: a T cell-mediated hypersensitivity reaction.

We highlight the importance of skin tests in IDT with late reading and/or patch test in the etiological diagnosis of DRESS, and the study of possible cross-reactivity with other drugs of the same group.

In conclusion, ICM are described in the literature as drugs that can induce a DRESS syndrome, although they are not a rare but infrequent cause of this reaction. IDT and/or patch skin tests allow the etiological diagnosis and the study of cross-reactivity, however the predictive values in severe skin reactions such as DRESS are not clear.

We present a case of DRESS syndrome due to ioversol with cross-reactivity to iomeprol demonstrated by positive IDT. In this case, excisional punch biopsy of the positive IDT was consistent with DRESS syndrome caused by T-lymphocyte activation, supporting the established clinical diagnosis.

## Data Availability

The authors confirm that the data supporting the findings of this clinical case are available within the article.

## Abbreviations

DRESS: Drug reaction with eosinophilia and systemic symptoms
SCAR: Svere cutaneous adverse drug reactions
NIHR: Non-inmediate hypersensitivity reactions
ICM: Iodinated contrast media
IDT: Intradermal test
